# Recent Advances in Enhancement Strategies for Osteogenic Differentiation of Mesenchymal Stem Cells in Bone Tissue Engineering

**DOI:** 10.3389/fcell.2022.824812

**Published:** 2022-02-23

**Authors:** Kangkang Zha, Yue Tian, Adriana C. Panayi, Bobin Mi, Guohui Liu

**Affiliations:** ^1^ Department of Orthopaedics, Union Hospital, Tongji Medical College, Huazhong University of Science and Technology, Wuhan, China; ^2^ Hubei Province Key Laboratory of Oral and Maxillofacial Development and Regeneration, Wuhan, China; ^3^ Department of Military Patient Management, The Second Medical Center & National Clinical Research Center for Geriatric Diseases, Institute of Orthopaedics, Chinese PLA General Hospital, Beijing, China; ^4^ Division of Plastic Surgery, Brigham and Women’s Hospital and Harvard Medical School, Boston, MA, United States

**Keywords:** mesenchymal stem cell, osteogenesis, bone defect, bone healing, tissue engineering

## Abstract

Although bone is an organ that displays potential for self-healing after damage, bone regeneration does not occur properly in some cases, and it is still a challenge to treat large bone defects. The development of bone tissue engineering provides a new approach to the treatment of bone defects. Among various cell types, mesenchymal stem cells (MSCs) represent one of the most promising seed cells in bone tissue engineering due to their functions of osteogenic differentiation, immunomodulation, and secretion of cytokines. Regulation of osteogenic differentiation of MSCs has become an area of extensive research over the past few years. This review provides an overview of recent research progress on enhancement strategies for MSC osteogenesis, including improvement in methods of cell origin selection, culture conditions, biophysical stimulation, crosstalk with macrophages and endothelial cells, and scaffolds. This is favorable for further understanding MSC osteogenesis and the development of MSC-based bone tissue engineering.

## Introduction

Bone is an important organ that serves a wide range of functions, including preserving vital internal organs and structures, providing the levers for muscles, maintaining mineral homeostasis, secreting growth factors and cytokines, and providing the environment for hematopoietic cell development (Clarke, 2008). It is mainly comprised of osteocytes, osteoblasts, osteoclasts and extracellular matrix (ECM), which maintains a dynamic balance between bone resorption and bone formation (Yang and Liu, 2021). Bone is a vascularized organ that can undergo self-healing after less severe damage. However, it is still a challenge for orthopedists to treat large segmental bone defects (Gage et al., 2018). In addition, an increasing number of people are suffering osteoporosis as the population ages, in which bone quality is decreased and adversely affects the treatment of bone injury (Tarantino et al., 2011). Thus, the development of strategies for bone healing and regeneration represents an area that is of great significance to improve patients’ function and quality of life (Guda et al., 2014).

Over the past few decades, increasing attention has been given to bone tissue engineering for the treatment of bone damage. Multiple factors are essential in bone tissue engineering, such as an ideal microenvironment, appropriate scaffolds, and viable cell populations (Li J. J. et al., 2018; Zhao et al., 2020). Mesenchymal stem cells (MSCs) are adult stem cells with self-renewal, multiple differentiation and immunomodulation functions and are regarded as promising seed cells for bone tissue engineering (Seong et al., 2010; Wang et al., 2013). MSCs reside in a variety of tissues, such as bone marrow, peripheral blood, adipose tissue, umbilical cord, and placenta (Hass et al., 2011). MSCs are multipotent cells that are able to differentiate into a determined mesenchymal lineage under specific conditions, such as osteoblasts, chondrocytes, adipocytes, muscle cells, neural cells and keratinocytes (Han et al., 2019). The cell fate and differentiation direction of MSCs depend on various factors, including the cell origin and viability, extracellular environment, and physical stimulation (Chen et al., 2016; Halim et al., 2020). The identification of appropriate approaches that support the osteogenic differentiation of MSCs is important for bone tissue engineering.

Several clinical trials have proven that MSC-based bone tissue engineering is safe and effective in promoting bone healing and leading to functional outcomes in patients, but the long-term therapeutic effect cannot be guaranteed (Giannotti et al., 2013; Morrison et al., 2018; Garcia de Frutos et al., 2020). It has been proposed that MSCs contribute to bone healing through three different approaches: differentiation and replacement (Garg et al., 2017), secretion of cytokines and extracellular vesicles (Marolt Presen et al., 2019; Tsiapalis and O'Driscoll, 2020), and immunomodulatory activity (Medhat et al., 2019; Weiss and Dahlke, 2019). It is still difficult to judge which is the most important way for MSCs to improve bone regeneration. Nevertheless, the regulation of MSC osteogenesis is conducive to improving the therapeutic effect of MSC-based bone tissue engineering. How to make MSCs differentiate into osteocytes or osteoblasts and maintain their physiological function has become a field of extensive research.

**FIGURE 1:**
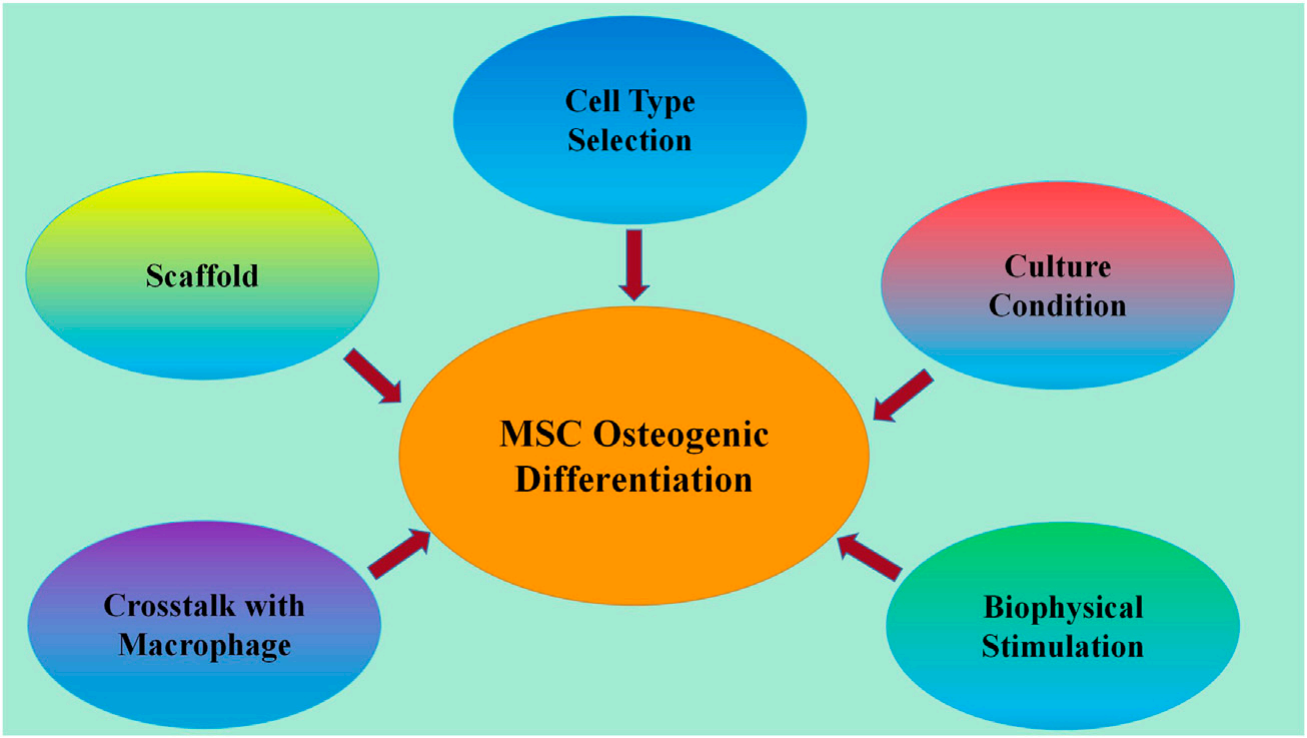
Developed methods for enhancing MSC osteogenic differentiation. Recent research progress on strategies for enhancing MSC osteogenic differentiation includes improvement of methods in cell origin selection, culture conditions, biophysical stimulation, crosstalk with macrophages and endothelial cells, and scaffolds.

In this review, we overviewed the recent research progress in enhancement strategies for MSC osteogenesis, including improvement of methods in cell origin selection, culture conditions, biophysical stimulation, crosstalk with macrophages and endothelial cells, and scaffolds (Figure 1). This will aid the further development of MSC-based bone tissue engineering.

## Osteogenic Differentiation of MSCs

**FIGURE 2:**
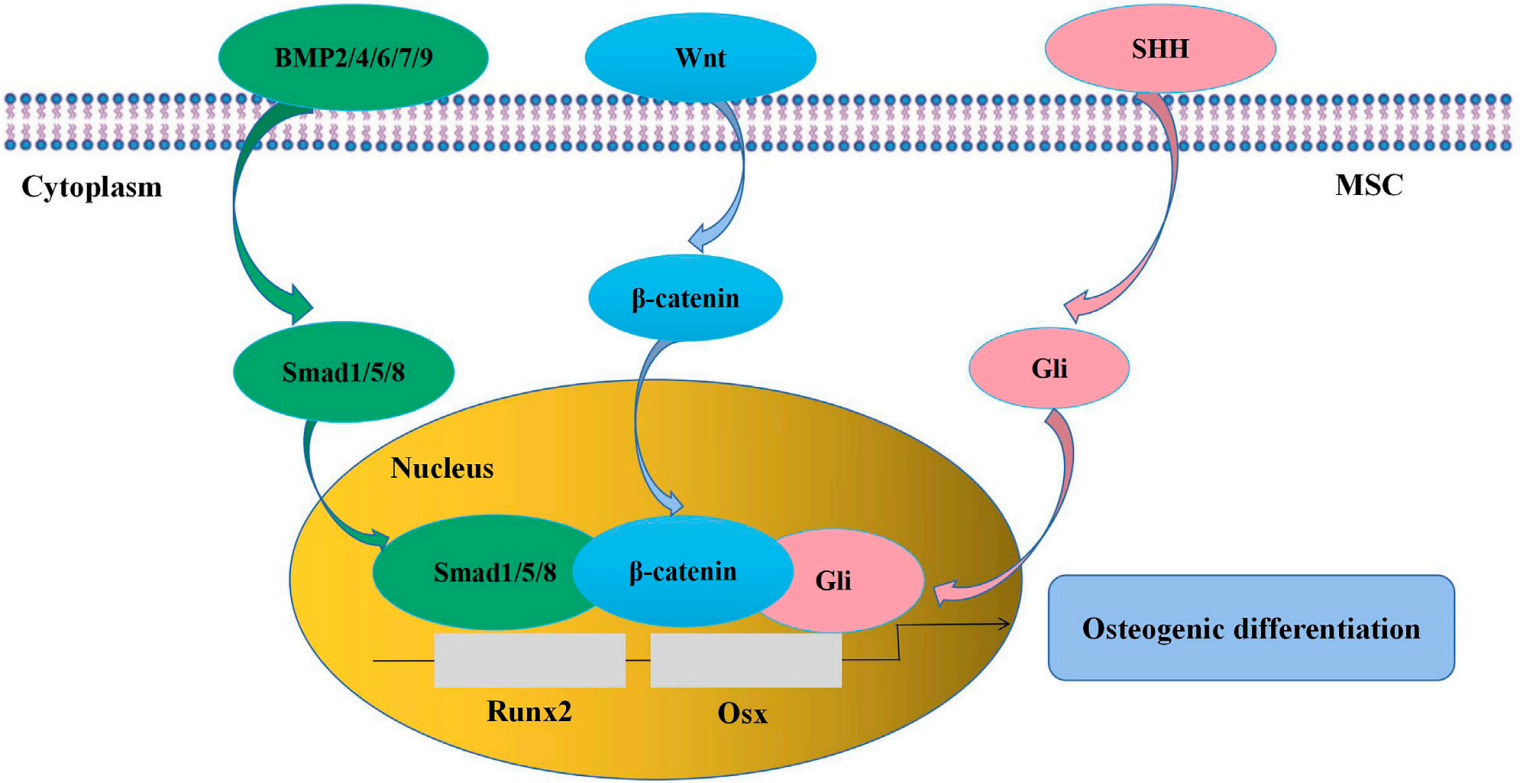
Signaling pathways in the regulation of MSC osteogenic differentiation. BMP signaling, Wnt signaling, and SHH signaling pathways are involved in the modulation of MSC osteogenesis, and the targets are the transcription factors Runx2 and Osx. MSC, mesenchymal stem cell; BMP, bone morphogenetic protein; Wnt, wingless-type MMTV integration site; SHH, sonic hedgehog; Runx2, runt-related transcription factor 2; Osx, osterix.

A thorough understanding of the regulation of MSC osteogenesis requires familiarity with the normal osteogenic differentiation process of MSCs. It is indicated that MSCs are prone to give rise to preosteoblasts for the first step instead of directly differentiating into osteocytes. Preosteoblasts develop into mature osteoblasts, which synthesize bone matrix and then become entombed in the matrix as osteocytes (James, 2013). The whole process is regulated by numerous signaling pathways, such as transforming growth factor-β (TGF-β)/bone morphogenetic protein (BMP) signaling, Wingless-type MMTV integration site (Wnt) signaling, and Sonic Hedgehog (SHH) signaling (Figure 2). As the targets of these signaling pathways, runt-related transcription factor 2 (Runx2) and osterix (Osx) are key transcription factors in the process of MSC osteogenic differentiation (Pokrovskaya et al., 2020). BMPs are members of the TGF-β superfamily, of which BMP-2 (Hu et al., 2017), -4 (Querques et al., 2019), -6 (Friedman et al., 2006), -7 (Kim Y. et al., 2018), and -9 (Wu et al., 2021) are involved in the promotion of MSC osteogenesis. BMP-2 is the most widely studied BMP in MSC osteogenic differentiation, and its function is achieved through the activation of downstream signaling, including in Drosophila mothers against decapentaplegic protein (Smad)1/5/8 (Li et al., 2014; Aquino-Martinez et al., 2017) and mitogen-activated protein kinase (MAPK) (Kong et al., 2012). Wnt signaling is considered another central signaling pathway in the regulation of MSC osteogenesis. The proosteogenic effect of Wnt signaling on MSCs can be achieved through both β-catenin-dependent and β-catenin-independent signaling pathways (Fakhry et al., 2013; James, 2013; Li Y. et al., 2018). It is reported that Wnt/β-catenin activity is involved in the regulation of bone development and bone remodeling (Little et al., 2002; Day et al., 2005; Chen and Long, 2013). Meanwhile, inactivation of Wnt/β-catenin in MSCs *in vitro* causes significant inhibition of osteogenic differentiation and promotion of adipogenic or chondrogenic differentiation, indicating that Wnt/β-catenin signaling is important in determining whether MSCs will differentiate toward osteoblasts (Day et al., 2005; Zhou et al., 2019). The SHH signaling pathway also has a well-established effect on MSC osteogenesis at an early stage *via* the activity of the Gli transcription factor (James, 2013). The addition of SHH protein significantly stimulated MSC osteogenic differentiation and reduced MSC adipogenic differentiation (James et al., 2012). Interestingly, SHH signaling and BMP-2 signaling can interact with each other and synergistically promote osteogenic differentiation by regulating Smad activity in the murine MSC line C3H10T1/2 (Spinella-Jaegle et al., 2001; Yuasa et al., 2002).

## Heterogeneity in MSC Osteogenic Differentiation Potential

The International Society for Cellular Therapy has provided the following standard criteria for human MSCs: 1) must be plastic-adherent in standard culture conditions; 2) must have the capacity to differentiate into adipocytes, osteoblasts and chondroblasts; and 3) must express CD105, CD73 and CD90 and lack the expression of CD45, CD34, CD14 or CD11b, CD79α or CD19 and HLA-DR (Dominici et al., 2006). In recent years, increasing research has identified that MSCs are heterogeneous populations. It is well acknowledged that MSCs from different individual donors and tissue sources have different biological properties (Wang and Han, 2019). Moreover, MSCs can be divided into different subpopulations according to their expression of cell surface markers, which also exhibit unique characteristics and cellular functions. Thus, the selection and utilization of superior MSCs is fundamental to improve the therapeutic effect of bone tissue engineering.

### Characteristics of Donors

The osteogenic differentiation potential of MSCs from donors of different ages has been studied. Gene expression analysis revealed that bone marrow-derived MSCs (BMSCs) from 3- and 6-month-old mice expressed similar levels of osteogenic differentiation-related genes (Bragdon et al., 2015). Tokalov et al. (2007) isolated BMSCs from rats of 2–48 weeks of age and reported that MSC osteogenesis was independent of donor age, as revealed by similar levels of calcium accumulation after osteogenic induction *in vitro*. Lee et al. (2021) demonstrated that the osteogenic differentiation potential of human BMSCs was not impaired in older donors, as shown by alizarin red staining. Similar results were found by Ding and his coworkers, who revealed that the osteogenic differentiation capacities of human adipose tissue-derived MSCs (ADSCs) between old age individuals and young age individuals were the same (Ding et al., 2013). These results suggest that MSC osteogenesis is independent of donor age, indicating that MSCs from elderly donors are eligible for bone tissue engineering in terms of osteogenic differentiation potential. However, Yang et al. (2014) analyzed the cellular properties of ADSCs isolated from 66 human donors (age: 10–70 years). Although they observed a trend in which the osteogenic differentiation ability of ADSCs declined as the donor age rose, it failed to reach statistical significance. Carvalho et al. (2021) found that the osteogenic differentiation potential of BMSCs from older fracture patients (60 and 80 years old) was inferior to that from younger fracture patients (30 and 45 years old), as evidenced by alkaline phosphatase (ALP) activity, calcium deposition, and osteogenic gene expression assays after 21 days under osteogenic differentiation conditions. The conflicting results obtained by these researchers might be due to the different cell sources, culture conditions, and evaluation methods used. The effect of donor age on the osteogenic differentiation capacity of MSCs remains controversial, and more related research is still needed.

Since bone formation and development are different between males and females, it is necessary to determine whether MSC osteogenesis was also sexually dimorphic. Leonardi et al. demonstrated that the osteogenic differentiation potential of human BMSCs was not affected by donor sex after 14 days of induction culture (Leonardi et al., 2008). Interestingly, Bragdon et al. (2015) revealed that the expression of bone-related genes in BMSCs derived from male mice and female mice was similar at 3 and 9 months, while at 6 months, BMSCs from female mice expressed these genes twofold greater than those from male mice. This suggests that at certain ages, MSC osteogenesis is different between males and females.

### Tissue Sources

In bone tissue engineering, bone marrow, adipose tissue, dental pulp, and umbilical cord are widely used as tissue sources of MSCs (Seong et al., 2010). The osteogenic differentiation abilities of MSCs from these tissues are heterogeneous. Above all, comparisons are often made between the osteogenesis of BMSCs and ADSCs. Lotfy et al. (2014) compared the characteristics of rat-derived BMSCs and ADSCs and found that BMSCs were more prone to differentiate into osteocytes after 2–3 weeks of induction culture than ADSCs. Similarly, Zaminy et al. (2008) studied the effects of melatonin on the osteogenic differentiation of rat-derived MSCs and concluded that BMSCs had greater potential for osteogenic differentiation than ADSCs, as determined by ALP activity and matrix mineralization assays. In addition, Lee et al. (2017) seeded dog-derived BMSCs and ADSCs on three-dimensional (3D)-printed polycaprolactone/tricalcium phosphate (PCL/TCP) scaffolds. When the composites were subjected to an *in vitro* osteogenic differentiation assay, the expression of genes associated with ossification was higher in BMSCs. These results indicate that BMSCs may represent a better candidate for bone tissue engineering than ADSCs regarding MSC osteogenesis. Dental pulp-derived MSCs (DPSCs), originating in the neural crest, are characterized by a fast proliferation rate and the capacity to differentiate into multiple cell lineages and have been widely used in the regeneration of periodontal bone defects (Ferro et al., 2014; Amghar-Maach et al., 2019; Lorusso et al., 2020). Pettersson et al. (2017) compared the osteogenic differentiation potential of DPSCs with jawbone-derived MSCs (JBMSCs) *in vitro* and reported no significant difference in osteogenesis between them. In other studies, it was demonstrated that DPSCs possessed a stronger ability to differentiate into osteoblasts than BMSCs both *in vitro* and *in vivo* (Ito et al., 2011; Jensen et al., 2016; Kumar et al., 2018). Wharton’s jelly derived MSCs (WJMSCs) appear to be another good choice for bone regeneration (Liu et al., 2017; Ansari et al., 2018; Kosinski et al., 2020). The osteogenic commitment in WJMSCs has been identified and was reported to be poorer than that in BMSCs and ADSCs (Zajdel et al., 2017; Cabrera-Perez et al., 2019). On the other hand, WJMSCs have reached a more advanced stage of immunomodulation action and proliferation ability, which deserves to be taken into account for bone tissue engineering (Kalaszczynska and Ferdyn, 2015; Vieira Paladino et al., 2019).

### Expression of Surface Markers

In recent years, increasing evidence has suggested that MSCs derived from the same tissue source express different surface markers, which reflect their different origins, statuses, and osteogenic differentiation potential (Table 1). CD73 is a well-known surface marker for MSCs in humans and mice. CD73^+^ mouse BMSCs were proposed to have increased “stemness” and greater osteogenic differentiation potential *in vitro* than CD73^−^ mouse BMSCs. When used to repair bone fractures in mice, CD73^+^ BMSCs also displayed an enhanced ability to promote fracture healing (Kimura et al., 2021). Gullo and De Bari (2013) used the combination of CD73 and CD39 (ectonucleoside triphosphate diphosphohydrolase 1, ENTPD1) to purify human synovial membrane-derived MSCs (SMSCs) and confirmed that CD73^+^CD39^+^ SMSCs exhibited significantly greater chondro-osteogenic potency than CD73^+^CD39^−^ SMSCs. CD200 is another potential new marker of BMSCs. Kim H. J. et al. (2018) evaluated the effect of CD200 on the cellular function of human BMSCs and found that CD200 overexpression significantly enhanced the osteogenic differentiation potential of BMSCs. In addition, Kouroupis et al. (2020) revealed that the expression of CD10 was associated with improved differentiation potential of human ADSCs. Ding et al. (2020) demonstrated that both CD10^+^ and CD10^−^ human adventitial cells exhibited phenotypic features of MSCs. Compared with their CD10^−^ counterparts, CD10^+^ adventitial cells showed higher proliferation ability and osteogenic differentiation potential. CD271, also known as low-affinity nerve growth factor receptor (LNGFR), has been regarded as an important surface protein of MSCs (Zha et al., 2021). Quirici et al. (2002) investigated the expression and function of CD271 in human BMSCs and demonstrated that CD271^+^ BMSCs exhibited greater CFU-F activity and adipogenic and osteogenic differentiation abilities, indicating that CD271 might be a “stemness” marker of BMSCs. Similar results were found in mouse and human ADSCs (Yamamoto et al., 2007; Barilani et al., 2018). However, it was challenged by Mikami and his colleagues, who found that the expression of CD271 could inhibit multipotential differentiation of DPSCs, including osteogenic differentiation (Mikami et al., 2011). These findings indicate that the effects of CD271 on different types of MSCs might be different or even opposite. In addition, CD271 expression is not consistently detectable in MSCs from fetal tissues, such as Wharton’s jelly, umbilical cord blood, and amniotic fluid, indicating that CD271 might not be an appropriate marker for the identification of functional subpopulations in fetal tissue-derived MSCs (Barilani et al., 2018). CD146, also known as melanoma cell adhesion molecule, is an adhesion molecule belonging to the immunoglobulin superfamily and is expressed in various types of MSCs. Ulrich et al. (2015) investigated the effect of CD146 on osteogenic differentiation of human placenta-derived MSCs (PDSCs) and demonstrated that CD146^+^ PDSCs had higher osteogenic differentiation and mineralized extracellular matrix production abilities than CD146^-^ PDSCs *in vitro*, indicating that CD146^+^ PDSCs might present a PDSC subpopulation that was predetermined to differentiate into osteoblasts. However, Paduano et al. (2016) found that CD146^Low^ human periapical cyst MSCs (PCy-MSCs) displayed stronger osteogenic differentiation potential than CD146^High^ PCy-MSCs. This variation might be attributed to the different types of MSCs they used. The role of CD146 in MSC osteogenesis requires more comprehensive and accurate research.

**TABLE 1:** Osteogenic differentiation potential different MSC subpopulations.

MSC subpopulations	Control	Species	Analysis methods	Results	References
BMSCs transfected with CD200	BMSCs transfected without interposed gene	human	ALP staining and gene expression and protein production of Runx2	CD200 expression increased the levels of ALP activity and Runx2 expression in BMSCs	Kim et al. (2018a)
CD73^+^ BMSCs	CD73^−^ BMSCs	mouse	Alizarin red staining, bone fracture repair *in vivo*	CD73^+^ BMSCs exhibited enhanced potentials for osteogenic differentiation *in vitro* and fracture repair *in vivo*	Kimura et al. (2021)
CD73^+^ CD39^+^	CD73^+^ CD39^−^ SMSCs	human	Alizarin red staining and expression of osteoblast genes	CD73^+^ CD39^+^	Gullo and De Bari, (2013)
SMSCs				SMSCs showed increase in calcium accumulation and gene expression of *Runx2*	
CD10^High^ ADSCs	ADSCs	human	Alizarin red staining	CD10^High^ ADSCs exhibited higher level of calcium accumulation	Kouroupis et al. (2020)
CD271^+^ BMSCs	PA BMSCs	human	Alizarin red S staining	CD271^+^ BMSCs had a larger mineralized area	Quirici et al. (2002)
CD271^+^ ADSCs	CD271^-^ ADSCs	mouse	Alizarin red S staining	CD271^+^ ADSCs were more prone to form calcium nodule after osteogenic differentiation	Yamamoto et al. (2007)
		human			Barilani et al. (2018)
CD271^+^ DPSCs	CD271^-^ DPSCs	human	ALP staining, Ca^2+^ level, and genes expression of *Runx2*, *Osterix*, *Osteocalcin*, and *Nestin*	ALP activity and Ca^2+^ levels were lower in CD271^+^ DPSCs; no difference in the expression level of osteogenic genes was detected	Mikami et al. (2011)
CD146^+^ PDSCs	CD146^-^ PDSCs	human	von Kossa staining	CD146^+^ PDSCs exhibited a higher level of spontaneous ossification	Ulrich et al. (2015)
CD146^Low^ PCy-MSCs	CD146^High^ PCy-MSCs	human	Alizarin red staining and expression of osteoblast genes	calcium accumulation and genes expression of *Runx2* and *Osteopontin* were greater in the CD146^Low^ than in CD146^High^ PCy-MSCs	Paduano et al. (2016)

*BMSCs*, bone marrow-derived mesenchymal stem cells; *ALP*, alkaline phosphatase; *SMSCs*, synovial membrane-derived mesenchymal stem cells; *ADSCs*, adipose tissue-derived mesenchymal stem cells, *PA*, plastic adherent; *DPSCs*, dental pulp-derived mesenchymal stem cells; *PDSCs*, placenta-derived mesenchymal stem cells; *PCy-MSCs*, periapical cyst mesenchymal stem cells.

## Culture Conditions

In general, MSCs isolated from tissues need to be cultured and expanded *in vitro* before *in vivo* transplantation. The improvement of culture conditions might be an efficient approach to enhance MSC osteogenesis. It is well recognized that conventional 2D culture is unable to mimic the *in vivo* 3D MSC niche, which is characterized by cell-cell and cell-ECM interactions. The drawback of 2D culturing methods has currently promoted the development of 3D MSC culture. In an effort to more closely recapitulate the *in vivo* microenvironment, both cellular properties and functions of MSCs, such as phenotype, differentiation ability and immunomodulatory action, can be preserved or enhanced by 3D culturing technologies (Kouroupis and Correa, 2021). Recent studies have compared the osteogenic differentiation abilities of MSCs in 2D monolayers and 3D culture systems. It was demonstrated that MSCs in 3D culture systems (e.g., scaffolds and microcarriers) exhibited spread morphology and were more prone to differentiate into osteoblasts than MSCs in 2D cultures, indicating that 3D cultures might be more suitable for bone tissue engineering (Brennan et al., 2015; Shekaran et al., 2015). In addition, flow perfusion culture has been shown to enhance osteoblastic differentiation and ECM deposition of MSCs compared to static culture (Holtorf et al., 2005). Mitra et al. (2017) cultured human BMSCs in macroporous scaffolds in direct perfusion bioreactors and found that continuous dynamic culture conditions could significantly promote BMSC osteogenic differentiation, as shown by enhanced osteogenic gene expression and ectopic bone formation. In addition, MSC 3D spheroids have shown increased osteogenic differential potential compared to monolayer cultured MSCs (Griffin et al., 2017; Kim et al., 2019). Saleh et al. (2016) revealed that Wnt signaling was activated in MSC spheroids but not 2D cultured MSCs during osteogenic differentiation. Interestingly, Sankar et al. (2019) used a 3D double strategy for osteogenic differentiation of human ADSC spheroids on patterned poly (lactic-co-glycolic acid) (PLGA)/collagen/hydroxyapatite (HA) electrospun fiber mats and found that the osteogenic differentiation of ADSCs was significantly enhanced even in the absence of osteogenic induction culture medium.

Several studies have shown that aged MSCs after long-term *in vitro* expansion exhibit decreased osteogenic differential potential (Yu et al., 2014; Bertolo et al., 2016; Yang et al., 2018). Senescence is associated with the impaired differentiation ability of late-passage MSCs, which show decreased colony-forming unit (CFU) activity, reduced proliferation capacity, and increased senescence-associated β-galactosidase activity and gene expression (Bertolo et al., 2016; Grotheer et al., 2021). Thus, it is suggested that MSCs at early passages are more appropriate candidates for bone tissue engineering. Oxidative stress is another factor that could impact the behaviors of MSCs, including their proliferation, differentiation and immunomodulation functions. Increased reactive oxygen species (ROS) usually promote MSC adipogenesis but impair MSC osteogenesis (Denu and Hematti, 2016). Binder et al. (2015) indicated that reduced serum (5%) and hypoxic conditions (5%) in culture medium could enhance osteogenic differentiation in human BMSCs. Similar effects of hypoxia were also found in human PDSCs (Gu et al., 2016) and ADSCs (Fotia et al., 2015). However, MSCs exposed to excessively low oxygen content (1%) demonstrated decreased osteogenic differentiation capacity, which is associated with increased expression of *hypoxia inducible factors* (*HIFs*) and *Notch1* (Tamama et al., 2011; Yang et al., 2019). In addition, it has been proposed that MSC osteogenesis is influenced by the glucose content in the culture medium. Aswamenakul et al. (2020) confirmed that human BMSC osteogenesis was reduced under high glucose conditions (10, 25, and 40 mM), as revealed by Alizarin red S staining and ALP activity assays.

## Biophysical Stimulation

Physical stimulation has been proposed to affect MSC fate and differentiation by initiating or strengthening biochemical signaling (Wang and Chen, 2013; Huang et al., 2015). The effects of mechanical stimulation, electric field, and electromagnetic field on MSC osteogenesis have been widely investigated over the past few years.

### Mechanical Stimulation

Since the promotion of exercise on bone repair and reconstruction in clinical settings is well recognized, the effect of mechanical stimulation on MSC osteogenesis is worth exploring. Hu et al. (2013) conducted a study in which they delivered noninvasive dynamic hydraulic stimulation (DHS) to rat mid-tibiae and found that BMSCs in the stimulated tibiae were induced into osteoblasts in a time-dependent manner. In addition, Gharibi et al. (2013) seeded human BMSCs onto calcium phosphate scaffolds and subjected the composite to an appropriate pulsating compressive force (5.5 ± 4.5 N at a frequency of 0.1 Hz). Gene expression analysis showed that *Runx2* was significantly upregulated after 22 h of loading. Kang et al. (2012) examined the impact of mechanical strain on the osteogenic differentiation of human umbilical cord-derived MSCs (UCMSCs) and revealed that mechanical strain (5% or 10% strain magnitude, 5 s of stretch and 15 s of relaxation) decreased the protein expression of MSC surface antigens, such as CD73, CD90, and CD105, while increasing the gene expression of osteogenic markers, such as *osteopontin* (*OPN*), *osteonectin* (*ON*), and *type I collagen (COL I)*. Similar results were reported by Li and his coworkers in rat BMSCs, who demonstrated that the gene expression of *Runx2* and *Osx* and the production of COL I were more strongly induced in cells subjected to mechanical strain (5% strain magnitude, 6 h/day, 10 times/min) compared to those in unstrained groups (Li et al., 2015). The underlying mechanism by which mechanical stimulation regulates MSC osteogenesis has been investigated. It has been proposed that cell–cell and cell-ECM adhesion is the major structure for MSCs to sense mechanical stimulation. Integrin is a transmembrane protein on MSCs and acts as a bridge between the ECM and intracellular actomyosin cytoskeleton in mechanical transmission, resulting in the activation of downstream signaling pathways (Sun et al., 2021). Qi et al. (2008) indicated that mechanical strain was able to promote BMSC osteogenesis through upregulation of the transcription factors core binding factor alpha 1 (Cbfa1) and v-ets erythroblastosis virus E26 oncogene homolog 1 (Ets-1). In addition, Chen et al. (2018) demonstrated that mechanical stretching could improve MSC osteogenic differentiation through activation of the AMP-activated protein kinase (AMPK)-silent information regulator type 1 (SIRT1) signaling pathway.

### Electrical Stimulation

Electrical stimulation has emerged as a useful tool to enhance MSC osteogenic differentiation and bone healing. It was found that exposing human BMSCs to an appropriate electrical current (10 or 40 mA, 10 Hz, sinusoidal waveform, 6 h/day) resulted in enhanced osteogenic differentiation, as evidenced by significantly increased expression of the osteogenic marker genes *Runx2*, *Osx*, *OPN* and *osteocalcin* (*OCN*) (Creecy et al., 2013). Similar findings were achieved by Zhang and his coworkers in human ADSCs (Zhang et al., 2018). Eischen-Loges et al. (2018) reported that treatment with electrical stimulation (100 mV/mm, 1 h/day) significantly promoted rat BMSC osteogenic differentiation, and this effect lasted a maximum of 7 days after electrical stimulation was discontinued. Furthermore, Leppik et al. (2018) combined ADSCs, β-TCP scaffolds and electrical stimulation (1.2 V, 80 mAh) to treat large bone defects in rats and found that bone healing was more strongly improved in the electrically stimulated group than in the control group. Interestingly, Hou et al. efficiently initiated the process of MSC osteogenic differentiation by optimizing electrical stimulation parameters based on the calcium spike patterns of MSCs (Hou et al., 2019). The effects of electrical stimulation on cellular properties and functions are known to be achieved through induction of conformational changes in voltage-sensitive proteins, reversible pore formation in plasma membranes, Ca^2+^ influx, and activation of various signaling pathways (Thrivikraman et al., 2018; Ning et al., 2019). It has been demonstrated that electric fields were able to induce activation of the wnt/β-catenin signaling pathway and BMP signaling pathway (Zhang et al., 2014; Kwon et al., 2016). However, how electrical cues are transferred into intracellular molecular signals that result in MSC osteogenic differentiation remains unclear and needs further investigation.

### Magnetic Stimulation

Magnetic stimulation is another physical approach to regulate MSC osteogenic differentiation. Kim et al. (2015) evaluated the effects of static magnetic field treatment on the osteogenic differentiation of human BMSCs. Their results demonstrated that a moderate intensity (15 mT) magnetic field promoted osteoblastic differentiation in BMSCs, as determined by increased ALP activity, mineralized nodule formation, calcium content, and expression of osteogenic markers, such as *Run×2*, *Osx*, *OCN*, *ON*, *OPN*, *COL I* and *bone sialoprotein 2* (*BSP2*) In another study, Ceccarelli et al. investigated the effects of pulse electromagnetic field (PEMF) (magnetic field intensity: 2—0.2 mT, electric tension amplitude: 5—1 mV, 75—2 Hz, pulse duration: ∼1.3 msec) exposure on the osteogenic differentiation of human BMSCs and ADSCs. Bone-related ECM deposition was more strongly induced in BMSCs than in ADSCs, indicating that the promoting effect of PEMFs might be more efficient in BMSCs (Ceccarelli et al., 2013). It has been proposed that cells respond to magnetic stimulation with changes in cytoskeleton remodeling, membrane potential, ion channel gating, and targeted gene expression (Zablotskii et al., 2018). However, the underlying mechanism by which magnetic stimulation promotes MSC osteogenic differentiation has not been revealed and needs to be studied in the future.

## Crosstalk With Macrophages and Endothelial Cells

Macrophages, key cells of innate immunity, can be found in nearly all tissues during inflammation and infection. The important roles of macrophages in the secretion of anti-inflammatory factors and the recruitment and regulation of the differentiation of MSCs during bone healing have been revealed in recent years (Pajarinen et al., 2019). In response to environmental signals, macrophages can undergo polarization into the M1 phenotype (related to the inflammatory response) and M2 phenotype (related to inflammation resolution and tissue regeneration) (Sinder et al., 2015; Pajarinen et al., 2019). Gong et al. utilized cocultures of mouse macrophages and BMSCs to investigate the effects of macrophages with different phenotypes on mediating MSC osteogenic differentiation. They found that osteogenic markers, ALP activity, and bone mineralization were increased in MSCs cocultured with M2 macrophages but decreased in MSCs cocultured with M1 macrophages. The effects might be regulated by M2 macrophage-derived pro-regenerative cytokines, such as TGF-β, VEGF, and IFG-1, and M1 macrophage-derived inflammatory cytokines, such as IL-6, IL-12, and TNF-α (Gong et al., 2016). Similar results were obtained by Zhang and his coworkers in human ADSCs (Zhang et al., 2017). It was suggested that the soluble proteins BMP-2, -6 and oncostatin M (OSM) produced by M2 macrophages and related signaling pathways might be involved in the promotion of MSC osteogenic differentiation (Zhang et al., 2017; Wang et al., 2021). In addition, Luo et al. (2020) indicated that macrophages stimulated BMSC osteogenesis by reducing intracellular ROS, which was increased during osteogenic differentiation. However, researchers found that in a 3D coculture system, both M1 and M2 macrophages inhibited the osteogenic differentiation of human ADSCs (Tang et al., 2019). The conflicting conclusions might be due to the use of different cell ratios, culture times and means, and polarization methods for macrophages. Therefore, the role of macrophages in the osteogenic differentiation of MSCs needs to be investigated more comprehensively and accurately.

It is well recognized that angiogenesis is mandatory for successful bone repair. The crosstalk between endothelial cells and MSCs has been studied in the past decade. The coculture of endothelial progenitor cells and MSCs is proposed to have a synergistic effect in terms of angiogenesis and bone formation, in which endothelial progenitor cells promote osteogenesis, and conversely, MSCs foster angiogenesis (Bouland et al., 2021). Gershovich et al. (2013) evaluated the effects of coculturing BMSCs and human umbilical vein endothelial cells on BMSC osteogenic differentiation and found that ALP activity, collagen production, and calcium nodule formation were significantly promoted. Chen et al. cocultured rabbit endothelial progenitor cells and peripheral blood-derived MSCs (PBSCs) on a 3D calcium phosphate bioceramic scaffold and found that the expression of osteogenic- and vascular-related genes was increased *in vitro*. When the cell-scaffold construct was used to repair large bone defects in rabbits, both new bone and promoted vascularization were observed *in vivo* (Chen et al., 2019). Similar results were obtained by Liang et al. (2016), who utilized cocultures of rat EPCs and BMSCs to treat alveolar bone defects in rats. The underlying mechanism by which endothelial cells regulate MSC osteogenic differentiation has been partly revealed. It has been proposed that endothelial cells directly interact with MSCs and regulate MSC osteogenesis *via* gap and adherence junctions (Bouland et al., 2021). In addition, endothelial cells can promote MSC osteogenic differentiation through the secretion of growth factors, such as BMP-2, endothelin-1 (ET-1), and insulin-like growth factor (IGF), which interact with specific membrane receptors on MSCs (Grellier et al., 2009). Xu et al. (2020) indicated that the MAPK signaling pathway was involved in the regulation of endothelial progenitor cells on MSC osteogenic differentiation. They found that silencing the expression of p38 resulted in decreased osteogenic gene expression, ALP activity, and calcium deposition in cocultured MSCs.

## Scaffold

The scaffold is an essential component of bone tissue regeneration, which supports MSC adhesion and survival by providing a 3D structure and forming the cell niche. In addition, both the composition and structure of scaffolds can regulate MSC fate and behaviors, such as cell migration, proliferation and differentiation (Garcia-Sanchez et al., 2019). Thus, culturing MSCs onto scaffolds may be an efficient approach to improve the engraftment of MSCs and the therapeutic effects of MSC-based bone tissue engineering. Designing an appropriate scaffold for bone healing has been a focus of research in bone tissue engineering, in which the stimulatory effect on MSC osteogenesis is an important aspect (Table 2).

**TABLE 2:** The effects of scaffolds on MSC osteogenic differentiation.

Aspects	Scaffold features	MSCs	Effects on MSC osteogenic differentiation	References
composition	nanoHA/collagen scaffold modified with phosphorylated amino acids	human BMSCs	BMSCs underwent osteogenic differentiation *in vitro* in the absence of osteogenic inductor and ectopic bone formation *in vivo*	Salgado et al. (2019)
	collagen/glycosaminoglycan scaffold incorporated with a calcium phosphate mineral phase	human BMSCs	the scaffold promoted osteogenic differentiation and mineral deposition of BMSCs within osteogenic induction media	Caliari and Harley, (2014)
	PCL scaffold coated with human BMSCs derived ECM	human BMSCs	BMSCs seeded on the scaffold exhibited an increase in calcium deposition and expression of bone-specific genes	Silva et al. (2020)
	gelatin scaffold incorporated with magnesium calcium phosphate	rat BMSCs	BMSCs exhibited enhanced osteogenic differentiation, as shown by increased ALP activity	Hussain et al. (2014)
	PLGA microspheres with tunable Mg^2+^ release	rat	the scaffold promoted BMSC osteogenic differentiation *in vitro* and resulted in significant bone regeneration in rats with critical-sized calvarial defects	Yuan et al. (2019)
		BMSCs		
structure	calcium phosphate scaffolds with hemispherical concavities of various sizes	human ADSCs	ADSCs seeded on scaffolds with 440 and 800 μm concavities, but not with 1800 μm concavities, showed enhanced osteogenic differentiation	Urquia Edreira et al. (2016)
	3D printed PPF porous scaffolds	human BMSCs	scaffolds with ordered cubic pores were more suitable for the promotion of BMSC osteogenic differentiation than that with cylindrical pores	Ferlin et al. (2016)
	3D printed PCL/DCM scaffolds with micro/nanosurface pores	human BMSCs	BMSCs displayed increased ALP activity and osteocalcin production in osteogenic medium	Prasopthum et al. (2019)
	barium titanate nanoparticle/alginate scaffold	human DPSCs	DPSCs exhibited higher levels of *BMP-2* and *ALP* genes expression	Amaral et al. (2019)
bioactive molecule delivery	chitosan oligosaccharide/heparin nanoparticles-modified chitosan-agarose-gelatin scaffold with sustainable BMP-2 release	mouse BMSCs	the scaffold induced BMSC differentiation towards osteoblasts in the absence of osteogenic media	Wang et al. (2018)
	titanium dioxide scaffold with alginate hydrogel containing simvastatin	human ADSCs	ADSCs seeded on the scaffold showed increased expression of osteogenic genes and proteins	Pullisaar et al. (2014)
	β-TCP scaffold containing human-induced pluripotent stem cell-derived MSC-derived exosomes	human BMSCs	the scaffold increased the levels of ALP activity and calcium deposition of BMSCs in osteogenic media	Zhang et al. (2016)

*HA*, hydroxyapatite; *BMSCs*, bone marrow-derived mesenchymal stem cells; *PCL*, polycaprolactone; ECM, extracellular matrix; *ALP*, alkaline phosphatase; PLGA, poly (lactic-co-glycolic acid), *ADSCs*, adipose tissue-derived mesenchymal stem cells; *PPF*, Poly Propylene Fumarate), *DCM*, dichloromethane; *DPSCs*, dental pulp-derived mesenchymal stem cells; *BMP-2*, bone morphogenetic protein-2, *β-TCP* β-tricalcium phosphate.

The composition is a key factor that needs to be taken into account when designing scaffolds to enhance MSC osteogenesis. Many different materials have been applied to fabricate scaffolds in bone tissue engineering, including natural and synthetic materials. Natural materials, such as collagen, ECM, calcium phosphate, chitosan, hyaluronic acid, silk fibroin and alginate, are widely used due to their high biocompatibility and biodegradability (Tang et al., 2021). Of these, collagen, ECM and calcium phosphate are probably most commonly used because of their abilities to replicate the properties of the bone microenvironment and to promote MSC osteogenic differentiation (Curry et al., 2016; Dong and Lv, 2016). For example, Salgado et al. (2019) developed an HA/collagen scaffold that was modified with phosphorylated amino acids. The results of ectopic bone formation analysis showed that the scaffold could promote osteogenic differentiation and bone-related ECM deposition of human BMSCs. In another study, Caliari and Harley (2014) endowed the collagen/glycosaminoglycan scaffold with the ability to promote osteogenic differentiation of human BMSCs by incorporation of a calcium phosphate mineral phase. However, their applications in bone tissue engineering are limited by unsatisfactory mechanical strength and rapid degradation rate. Thus, they are often used in conjunction with synthetic polymers, which possess low biocompatibility but sufficient mechanical strength. For example, Silva et al. (2020) coated human BMSC-derived ECM on a 3D polycaprolactone (PCL) scaffold and demonstrated that the composite scaffold was able to modulate BMSC behavior in favor of differentiation into osteoblasts. Recently, the application of biodegradable metals and their alloys has shown broad prospects in bone fracture healing. Increasing evidence demonstrates that calcium (Ca) and magnesium (Mg) ions are able to promote the osteogenic differentiation of MSCs (Park et al., 2019; Hohenbild et al., 2021). Hussain et al. (2014) found that rat BMSCs seeded on gelatin scaffolds incorporating magnesium calcium phosphate exhibited enhanced osteogenic differentiation, as shown by increased ALP activity. Yuan et al. (2019) developed injectable PLGA microspheres with tunable Mg^2+^ release and confirmed that they were able to promote rat BMSC osteogenic differentiation *in vitro* and result in significant bone regeneration *in vivo*.

In addition, the microstructure of the scaffold is also proposed to have an impact on the osteogenic differentiation of MSCs. The porosity and appropriate pore size of the scaffold were considered influencing factors for MSC osteogenesis (Kasten et al., 2008). Urquia Edreira et al. (2016) conducted a study in which they cultured human ADSCs on calcium phosphate scaffolds with hemispherical concavities of various sizes (440, 800 or 1800 μm). They revealed that ADSCs seeded on scaffolds with 440 and 800 μm concavities, but not with 1800 μm concavities, exhibited enhanced osteogenic differentiation. Ferlin et al. (2016) investigated the impact of pore geometries on human BMSC osteogenic differentiation and found that osteogenic marker expression at early timepoints was increased in BMSCs cultured on scaffolds with cylindrical pores, while BMSCs cultured in scaffolds with ordered cubic pores expressed late osteogenic markers, suggesting that ordered cubic pores might be more suitable for the promotion of MSC osteogenic differentiation. However, the underlying mechanism is not fully understood and needs further investigation. In addition, based on the advancement of manufacturing technology, 3D printing technology has been applied to fabricate porous scaffolds with 3D architecture, good biocompatibility, and bone induction function (Wang et al., 2020). For example, Prasopthum et al. fabricated 3D printed polymer scaffolds with micro/nanosurface pores (0.2–2.4 μm) and found that they were able to promote human BMSC osteogenic differentiation in the absence of soluble differentiation factors (Prasopthum et al., 2019). Recently, the application of nanomaterial-based scaffolds in bone tissue engineering has also received much attention, showing improved bone regeneration effects compared with conventional scaffolds. It has been proposed that nanomaterials can promote MSC osteogenic differentiation due to their specific chemical, physical and mechanical properties (Zhang et al., 2021). The commonly used nanomaterials in bone tissue engineering include metals and their derivatives, bioactive ceramics, carbon nanomaterials and polymers (Ye et al., 2020). For example, Las Amaral et al. (2019) designed a barium titanate nanoparticle/alginate scaffold that exhibited highly interconnected pores and surface nanotopography. The osteogenic differentiation of human DPSCs seeded on it was enhanced, as indicated by upregulated gene expression of BMP-2 and ALP.

Another strategy for inducing MSCs into osteoblasts is to design scaffolds containing spatially graded bioactive molecules. For example, Wang et al. (2018) constructed a chitosan-agarose-gelatin scaffold that was modified with chitosan oligosaccharide/heparin nanoparticles, which could sustainably release BMP-2 and induce mouse BMSC differentiation towards osteoblasts*.* Pullisaar et al. (2014) coated a titanium dioxide (TiO_2_) scaffold with alginate hydrogel containing simvastatin and found that human ADSCs seeded on it were more strongly induced into osteoblasts, as demonstrated by increased expression of osteogenic genes and proteins, compared with TiO_2_ scaffolds without simvastatin. Recently, exosomes have been introduced into bone tissue engineering, which also shows an osteogenic induction effect on MSCs (Qi et al., 2016; Yahao and Xinjia, 2021). Zhang et al. (2016) loaded human-induced pluripotent stem cell-derived MSC-derived exosomes on β-TCP scaffolds and confirmed that the composite was able to efficiently enhance the osteogenic differentiation of human BMSCs.

## Conclusion and Perspective

MSCs represent one of the most promising cell types in bone tissue engineering, in which researchers are always making efforts to guide MSCs to efficiently differentiate toward osteoblasts. In the present review, we provide an overview of recently developed strategies for enhancing osteogenic differentiation of MSCs, including selection of optimal cell origin, improvement of culture conditions, application of biophysical stimulations, crosstalk with M2 macrophages and endothelial cells, and fabrication of appropriate scaffolds. Although significant advances in the development of methods for promotion of MSC osteogenic differentiation have been achieved, there are still some issues that need to be resolved. First, numerous strategies display positive effects in promoting MSC osteogenic differentiation. However, the efficiency of different methods has not yet been compared. In addition, the safety and ease of applying these approaches also need to be considered before making a choice. Second, the in vivo microenvironment is quite different from that in vitro. Thus, the efficiency and safety of these methods should be evaluated in vivo. Third, the underlying mechanisms by which several methods regulate MSC osteogenic differentiation, such as how the presence of macrophages and magnetic fields increase MSC osteogenesis, remain unclear. Future research should focus on the signaling pathways leading to the response of MSCs to osteogenic stimulation.
